# Tailorable 2D MoS_2_ via Oxide Sulfidation for Photodetection and Contact Engineering

**DOI:** 10.3390/s26113523

**Published:** 2026-06-02

**Authors:** Chieh-Yu Kuan, Sheng-Po Chang, Shoou-Jinn Chang, Jone-Fang Chen, Wei-Chih Lai

**Affiliations:** 1 Department of Electrical Engineering, Institute of Microelectronics, National Cheng Kung University, Tainan City 70101, Taiwan; ch880628123@gmail.com (C.-Y.K.); jfchen@mail.ncku.edu.tw (J.-F.C.); 2 Department of Microelectronics Engineering, National Kaohsiung University of Science and Technology, Kaohsiung City 88157, Taiwan; 3 Department of Photonics, National Cheng Kung University, Tainan City 70101, Taiwan; weilai@mail.ncku.edu.tw

**Keywords:** two-dimensional semiconductors, MoS_2_, thickness-tunable, negative photoconductivity, contact engineering

## Abstract

**Highlights:**

**What are the main findings?**

- Controllable Oxide-to-Sulfide Synthesis: A scalable fabrication strategy was developed using RF magnetron sputtering of MoO_3_ precursors followed by precise chemical-vapor sulfidation to produce high-quality, thickness-tunable MoS_2_ films with uniform interlayer spacing.
- Environment-Induced Negative Photoconductivity: The research identified the emergence of negative photoconductivity in bilayer MoS_2_ under ambient conditions, attributed to electron-trapping states from molecular adsorption and surface oxidation rather than structural traps.

**What are the implications of the main findings?**

- Contact Engineering for Performance Enhancement: Low-temperature thermal annealing of Ni electrodes effectively optimizes the metal-semiconductor interface and improves effective carrier injection, significantly enhancing the photocurrent of 2D optoelectronic devices.
- Unified Design Strategy for 2D Sensors: These findings provide critical insights into how surface adsorption and interfacial properties govern carrier transport, offering a robust framework for engineering high-performance and scalable TMD-based sensing technologies.

**Abstract:**

To address contact-limited transport commonly encountered in two-dimensional semiconductors, this study fabricated few-layer two-dimensional molybdenum disulfide (MoS_2_) films on sapphire substrates via controllable oxide-to-sulfide conversion. Combined sputtering deposition of molybdenum trioxide and precise chemical-vapor sulfidation afforded high-quality, high-uniformity, and thickness-tunable MoS_2_. The resulting films exhibit distinct differences in the frequencies of the Raman modes, consistent elemental ratios, and uniform interlayer spacing of ~0.65 nm. The MoS_2_-based devices exhibit robust photodetection with microampere-scale photocurrents. Bilayer MoS_2_ exhibited negative photoconductivity under ambient atmosphere, which is hypothesized to be linked to environment-induced surface doping and molecular adsorption rather than permanent structural traps. Contact engineering via mild thermal annealing of Ni electrodes significantly enhanced the photocurrent by improving effective interfacial carrier injection. These findings underscore the oxide sulfidation strategy as a scalable approach for engineering the layer-dependent behavior of transition metal dichalcogenides for optoelectronic applications.

## 1. Introduction

The thickness of two-dimensional (2D) materials can be reduced to a single or a few atomic layers, resulting in unique physical, chemical, and electronic properties. The groundbreaking isolation of graphene in 2004 by Geim and Novoselov marked a pivotal moment in materials science, ushering in the era of 2D materials [1]. This discovery not only won them the Nobel Prize in Physics in 2010 but also ignited extensive research into other 2D materials, including transition metal dichalcogenides (TMDs), which have since become central to advancements in the next generation of semiconductor devices [2].

TMDs, exemplified by molybdenum disulfide (MoS_2_), tungsten disulfide (WS_2_), MoSe_2_, WSe_2_, MoTe_2_, WTe_2_, NbS_2_, NbSe_2_, and TaS_2_ [3,4,5], are layered materials with the chemical formula MX_2_ (M is a transition metal: Mo, W, Ti, Ta, or Nb, and X is a chalcogen element such as S, Se, or Te). The structure of these materials comprises strong in-plane covalent bonds and weak out-of-plane van der Waals interactions, enabling their exfoliation into monolayers having direct bandgaps ranging from 1 to 2 eV, high optical absorption, and strong light-matter interactions, making them highly attractive for electronic, optoelectronic, and sensing applications [6,7,8,9].

MoS_2_ and WS_2_ have emerged as the most widely studied and practically relevant TMDs. MoS_2_ exhibits high electron mobility, a tunable bandgap (~1.2–1.8 eV depending on the layer number), and strong photoluminescence, making it advantageous for photodetectors, field-effect transistors, and chemical sensors. Recent reports have further shown that MoS_2_ photoconductivity and conductance can be strongly affected by adsorbates, temperature, pressure, and chemical-vapor exposure, highlighting the importance of surface-controlled carrier transport in MoS_2_ devices [7,8]. WS_2_-based devices can achieve improved durability and reliability arising from the slightly larger bandgap (~1.3–2.0 eV) and excellent chemical and mechanical stability of WS_2_, especially in harsh environments [9,10]. High-quality monolayers and few-layer films of both materials, which are highly suitable for experimental studies, can be obtained using scalable methods such as chemical vapor deposition (CVD) and physical vapor deposition.

The synthesis and characterization of monolayer TMDs have been extensively studied, driven by their theoretically exceptional carrier mobility and high optical responsivity. However, the practical realization of large-area, high-quality monolayers remains extremely challenging. Issues such as precise thickness control, defect formation (e.g., sulfur vacancies and grain boundaries), substrate mismatch, film discontinuity, and transfer-related contamination degrade the electronic and optical performance of monolayer TMD devices [11]. Consequently, these devices often exhibit unstable current transport, high contact resistance, and poor reproducibility, limiting their scalability for real-world applications.

Recent studies have indicated a paradigm shift from an exclusive focus on monolayers to bilayer and few-layer TMDs. The synthesis of bilayer and few-layer films having higher crystallinity and improved continuity is relatively facile. Moreover, interlayer coupling enhances charge transport in these materials by reducing defect scattering. For instance, bilayer WS_2_ contacts significantly reduce contact resistance in MOSFETs, enabling stable operation for over one year [10]. Similarly, lateral heterostructures based on bilayer WS_2_–MoS_2_ and WS_2_–WSe_2_ exhibit superior photoresponsivity and faster response times compared to monolayer devices [12]. In gas sensing, few-layer MoS_2_ films or hybrid nanostructures demonstrate higher sensitivity and faster recovery owing to their enhanced conductivity and adsorption stability [13]. Furthermore, benchmarking studies of large-area monolayer MoS_2_ and WS_2_ field-effect transistors reveal mobility values of 30–40 cm^2^V^−1^s^−1^, despite significant device-to-device variability, emphasizing the practical advantages of few-layer films [11].

In this work, we focus on the coupled roles of film thickness, ambient surface adsorption, and metal-semiconductor interfaces in determining the performance of few-layer MoS_2_ photodetectors. The main contribution is not the isolated use of oxide sulfidation, MSM photodetectors, or annealed contacts, but the integration of these elements into a single thickness-tunable MoS_2_ platform. By systematically comparing monolayer, bilayer, and trilayer films, examining environment-induced negative photoconductivity, and evaluating annealing-induced improvement in carrier injection, this study provides practical insight into how surface and interface effects govern the photoresponse of scalable two-dimensional optoelectronic devices.

## 2. Materials and Methods

### 2.1. Substrate Preparation

To achieve the growth of large-area and high-quality 2D MoS_2_ and WS_2_ thin films, sapphire substrates were selected owing to their close lattice match with the target materials as well as their excellent thermal stability. The substrates were cut from 2-inch sapphire wafers into ~1 cm^2^ pieces with a thickness of 0.43 mm using a precision diamond blade. Prior to depositing the film, the cleaning process consisted of sequential ultrasonic baths in acetone, isopropanol, and deionized water for 5 min each to remove organic residues. The substrates were subsequently dried under a nitrogen flow and baked to eliminate residual moisture.

### 2.2. Film Growth

**Figure 1:**
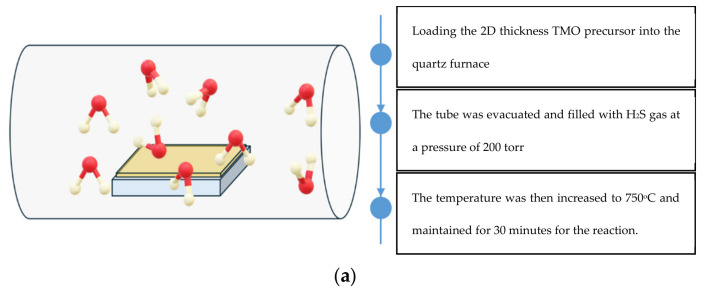

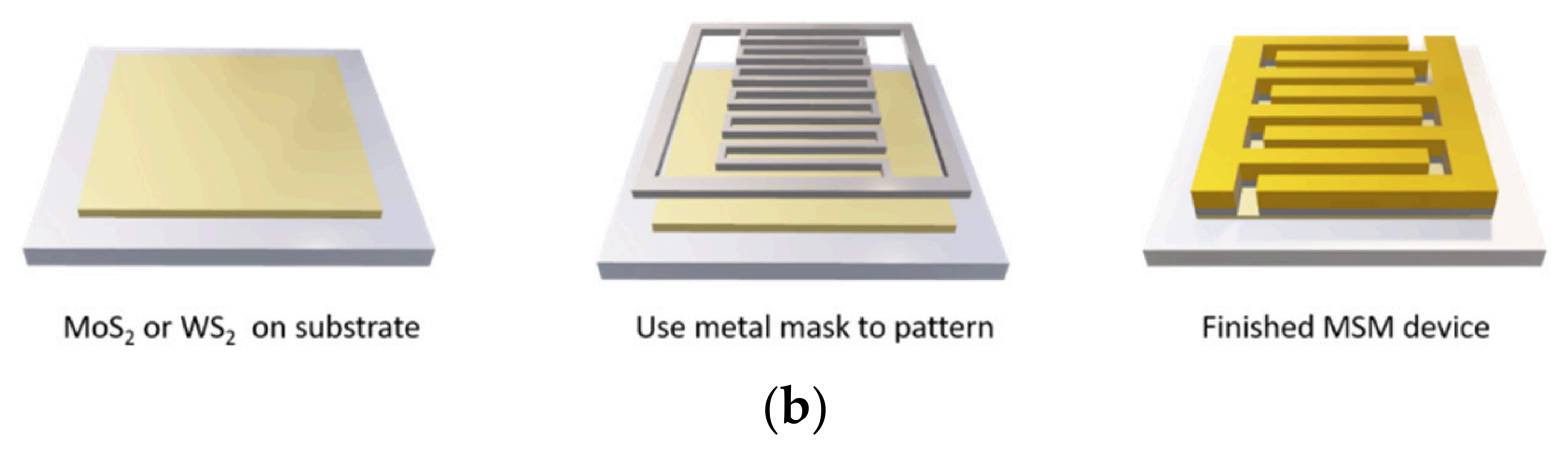
(**a**) Schematic diagram of the oxide-sulfidation process for few-layer TMD growth. (**b**) Schematic of the fabricated MSM photodetector with IDEs.

A combination of radio frequency (RF) magnetron sputtering and CVD was used for controllable synthesis. The film thickness was regulated by adjusting the deposition time of the transition metal oxide (TMO) precursor film. During sputtering, the deposition rate was minimized by operating under stable plasma conditions at a low RF power of 20 W. A sputtering duration of ~90 s yielded the monolayer MoS_2_ precursor. The oxide precursors were subsequently sulfurized in a quartz tube furnace. The schematic of the oxide-to-sulfide conversion process is illustrated in Figure 1a. Safety Note: Due to the high toxicity and flammability of H_2_S gas, the sulfurization process was strictly conducted in a well-ventilated setup equipped with appropriate exhaust scrubbing systems. The tube was evacuated to minimize exposure to oxygen, followed by the introduction of H_2_S gas at a constant pressure of 200 Torr. The reaction proceeded at 750 °C for 30 min, after which MoS_2_ or WS_2_ films were obtained.

### 2.3. Device Fabrication

To evaluate the optical responses of the films, metal-semiconductor-metal (MSM) devices with interdigitated electrodes (IDEs) were fabricated. A bilayer electrode structure consisting of 30 nm Ni or Ti (serving as the contact layer) and 70 nm Au (as the protective overlayer) was deposited via thermal evaporation using a shadow mask. To further enhance performance and mitigate contact-related limitations, contact engineering was conducted using the bilayer MoS_2_ photodetector. The device was subjected to short-duration, low-temperature annealing in a vacuum environment (100–300 °C for 30–60 min) to optimize the metal-semiconductor interface. The typical MSM device architecture and IDE configuration are presented in Figure 1b.

### 2.4. Characterization

Electrical measurements were conducted at room temperature using a semiconductor parameter analyzer (B1500A, Agilent Technologies Inc., Santa Clara, CA, USA) in a dark environment and under controlled illumination. Film properties were assessed using Raman spectroscopy (LabRAM HR, HORIBA Jobin Yvon SAS, Villeneuve d’Ascq, France, 532 nm excitation laser), atomic force microscopy (AFM, NT-MDT Co., Moscow, Russia), transmission electron microscopy ((JEM-2100F, JEOL Ltd., Akishima, Tokyo, Japan), optical microscopy (OM, Motic China Group Co., Ltd., Xiamen, China), and second-harmonic generation (SHG) imaging. Energy-dispersive spectroscopy (EDS, JSM-7001F, JEOL Ltd., Akishima, Tokyo, Japan) combined with TEM was further employed to verify film composition and local uniformity.

## 3. Results and Discussion

### 3.1. Material Analysis

The chemical-vapor sulfurization of transition metal oxides is represented by Equation (1):

(1) $$TMO\;+{3H}_{2}S\overset{∆}{\to }TMD\;+\;{3H}_{2}O\;+\;S$$

In Equation (1), Δ above the reaction arrow denotes the thermal energy supplied during the high-temperature sulfurization process. Under appropriate hydrogen sulfide flow and high-temperature conditions, the oxide film can be fully converted to a high-quality transition metal dichalcogenide film [14]. MoS_2_ thin films with controllable layer numbers were fabricated by the precise sputtering of a molybdenum trioxide(MoO_3_) precursor layer, followed by thermal sulfurization in a quartz tube furnace. The number of MoS_2_ layers and crystallinity were modulated by tuning the thickness and oxidation state of the MoO_3_ film. The overall sulfurization mechanism and pathways have been widely verified in previous studies [9,14,15,16].

For Raman spectroscopy, measurements were performed with a 532 nm excitation laser and 1800 gr/mm grating, providing a spectral resolution of approximately 0.3 cm^−1^. The laser spot size was approximately 1 μm, with an incident power maintained at 8 mW to prevent sample damage. Each spectrum was collected across the range of 350 cm^−1^ to 450 cm^−1^ using an acquisition time of 10 s, with 5 accumulations per measurement to ensure data reliability and consistency across all samples.

**Figure 2:**
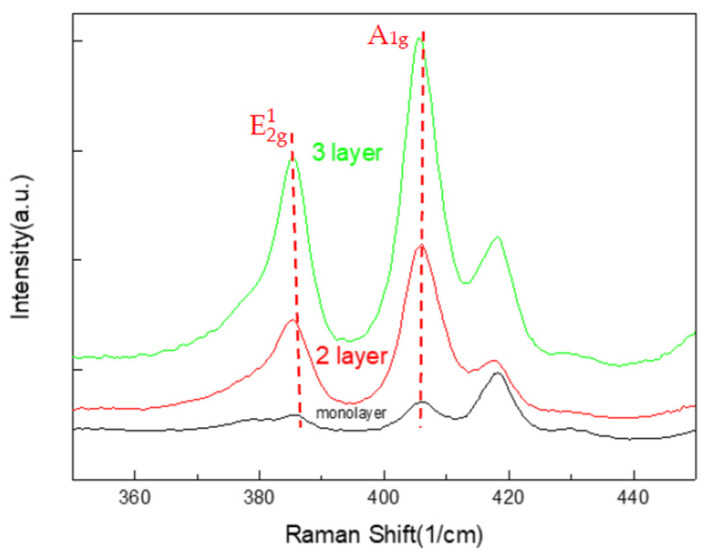
Raman spectra of MoS_2_ films with different layer numbers showing characteristic E_2_g^1^ and A_1_g peaks.

The Raman spectra of the MoS_2_ thin films of varying thicknesses (Figure 2) show two distinct characteristic phonon modes, E_2_g^1^ (~386.36 cm^−1^) and A_1_g (~405.55 cm^−1^), confirming the formation of MoS_2_. The frequency difference Δ(A_1_g − E_2_g^1^) increases with layer number, consistent with previous reports [17]. For monolayer MoS_2_, the difference of ~19 cm^−1^ indicates weak interlayer coupling. Table 1 summarizes the characteristic phonon-mode peaks and the corresponding peak separations for different layers of MoS_2_.

It can be observed that the Raman peak intensity increases with layer number. Therefore, it is inferred that the layer thickness significantly influences film quality. Although Raman intensity alone cannot directly represent the crystallinity of the films due to its dependence on measurement conditions, the observed trend under identical conditions, together with the OM and SHG imaging results, strongly supports the pronounced thickness-dependent continuity and uniformity of the MoS_2_ films.

**Figure 3:**
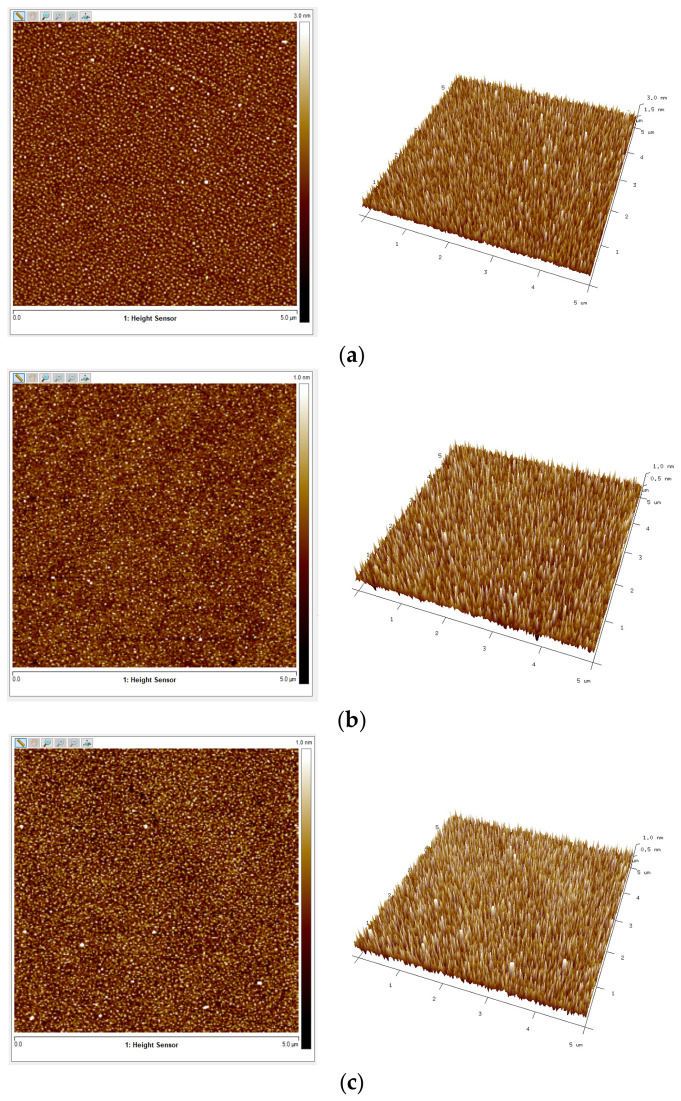
AFM topography analysis of (**a**) monolayer, (**b**) bilayer, and (**c**) trilayer MoS_2_ films.

AFM measurements were further performed to evaluate the surface morphology and roughness of monolayer, bilayer, and trilayer MoS_2_ films (Figure 3). Here, Rq represents the root-mean-square roughness, which is sensitive to larger height deviations. In contrast, Ra represents the arithmetic average roughness, which describes the average absolute surface-height deviation from the mean plane. The measured Rq/Ra values were 0.34/0.24 nm for monolayer MoS_2_, 0.131/0.0856 nm for bilayer MoS_2_, and 0.209/0.114 nm for trilayer MoS_2_, respectively. All Rq and Ra values are smaller than the ~0.65 nm thickness/interlayer spacing of monolayer MoS_2_, confirming the smooth surface morphology and high thin-film quality of the oxide-sulfidized samples. Notably, the monolayer film exhibits slightly higher roughness than the bilayer and trilayer films, consistent with the more pronounced island-like domains and grain boundaries observed in monolayer samples. The lower roughness of the bilayer and trilayer films indicates that MoS_2_ films with two or more layers exhibit improved continuity and surface quality compared with the monolayer film.

**Figure 4:**
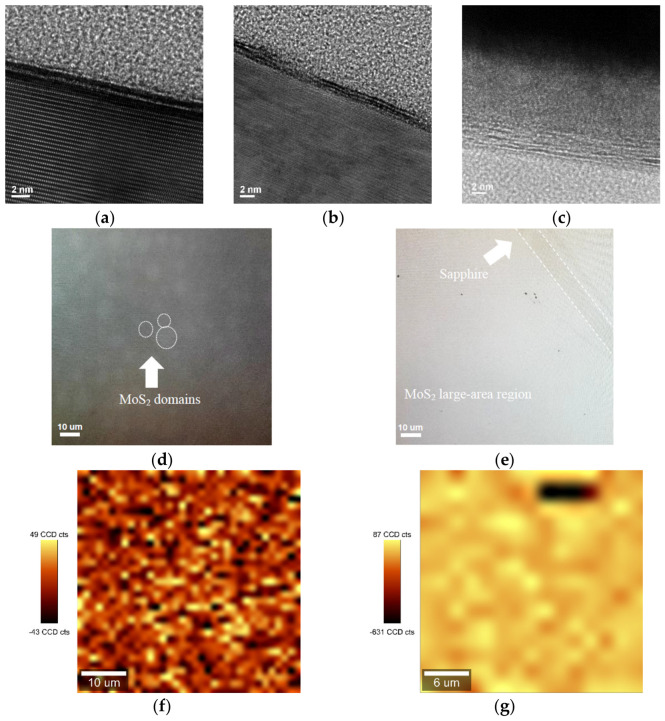
Microscopic and optical analysis results. Cross-sectional TEM image of (**a**) monolayer, (**b**) bilayer, and (**c**) few-layer MoS_2_ on sapphire. OM image of (**d**) monolayer and (**e**) trilayer MoS_2_ on sapphire. SHG image of (**f**) monolayer and (**g**) trilayer MoS_2_ on sapphire.

As shown in Figure 4a, cross-sectional TEM images of monolayer MoS_2_ revealed atomically ordered lattice fringes with a uniform interlayer spacing of approximately 0.65 nm, consistent with the typical d-spacing of MoS_2_ [18]. For further analysis, cross-sectional samples of bilayer and few-layer MoS_2_ were prepared, and TEM analysis is shown in Figure 4b,c, verifying that the number of layers can indeed be controlled by adjusting the thickness of the initial oxide precursor. These cross-sectional TEM observations provide direct thickness verification complementary to the Raman peak-separation analysis.

To verify the quality differences between monolayer and multilayer MoS_2_ films synthesized via oxide sulfurization and to account for the odd-layer requirement for SHG measurements, monolayer and trilayer MoS_2_ samples were subsequently prepared and characterized by OM and SHG analyses.

Figure 4d,e present the OM images of monolayer and trilayer MoS_2_ films grown on sapphire, respectively. The brighter regions correspond to the MoS_2_ films, while the darker regions represent the sapphire substrate. In the monolayer sample, numerous small MoS_2_ domains and pronounced grain boundaries are clearly visible, forming island-like features. This result indicates that monolayer MoS_2_ is relatively difficult to grow into a continuous large-area film. In contrast, the trilayer MoS_2_ sample exhibits a more uniform and continuous film morphology over a larger area, suggesting that thicker MoS_2_ layers facilitate the formation of large-area, continuous films.

Figure 4f,g show the SHG mapping images of monolayer and trilayer MoS_2_ on sapphire, respectively. The bright and dark regions indicate the presence and absence of detectable MoS_2_ films, respectively. Consistent with the OM observations, the SHG results further confirm that multilayer MoS_2_ exhibits improved large-area film continuity compared with monolayer MoS_2_.

Although monolayer films are difficult to grow over large areas, few-layer films exhibit excellent continuity and quality, as confirmed by OM and SHG comparisons.

**Figure 5:**
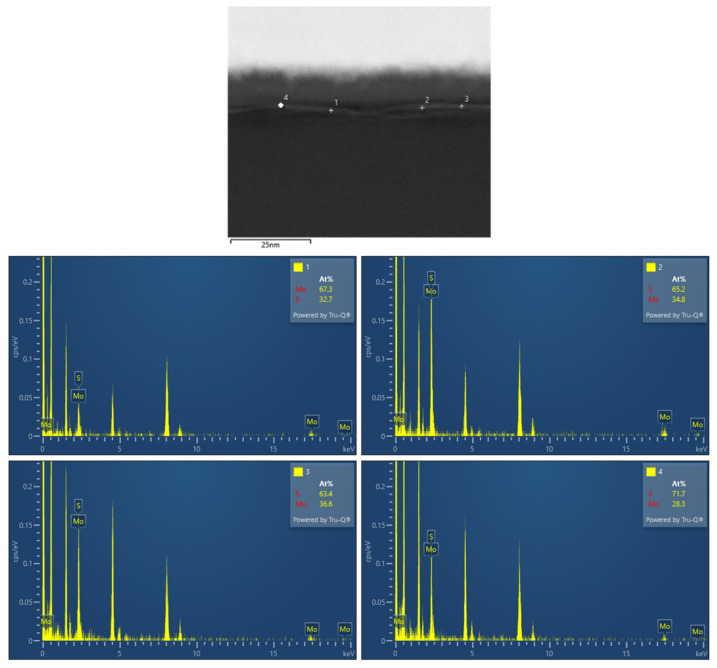
EDS atomic percentages of a representative bilayer MoS_2_ sample measured at four different positions.

EDS analysis of the elemental ratios (Figure 5) was performed at four different locations on a representative bilayer MoS_2_ sample. The bilayer sample was selected because it exhibited the best photodetection performance among the three layer numbers and therefore served as the key device structure for subsequent analysis. The measured Mo/S ratios were consistent with the stoichiometry of MoS_2_, confirming successful sulfurization in the analyzed film. Notably, all four spatially separated regions exhibited highly similar elemental distributions, indicating good local compositional uniformity. Because the monolayer, bilayer, and trilayer films were prepared using the same oxide-sulfidation chemistry and their layer numbers were independently verified by Raman, TEM, and AFM analyses, the bilayer EDS result supports the compositional uniformity and conversion quality of the oxide-sulfidized MoS_2_ films while avoiding overreliance on EDS as the sole evidence for all layer numbers.

### 3.2. Device Characterization

**Figure 6:**
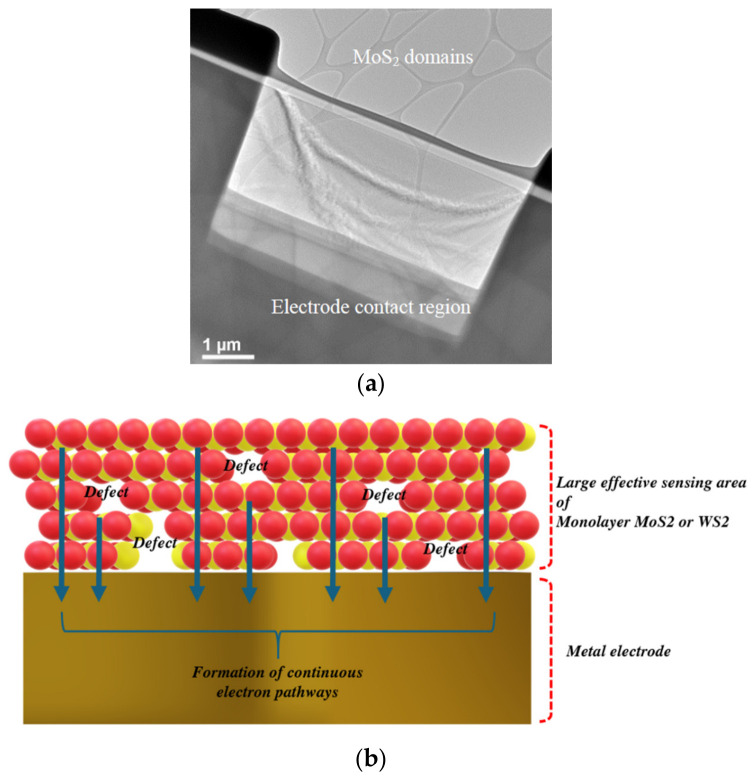
(**a**) Top view TEM image of a monolayer MoS_2_ sensor device. (**b**) Schematic of conductive pathway in a large effective sensing area configuration.

When applying these ultra-thin films to practical macroscopic devices, film continuity becomes a critical factor. Although monolayer films generally exhibit discontinuous grain domains and higher defect densities than few-layer structures, as shown in Figure 6a, their deployment in architectures with large effective sensing areas―such as electrolyte-gated field-effect transistor [9] configurations―can mitigate these transport bottlenecks. As schematically illustrated in Figure 6b, a large contact area facilitates the formation of continuous electron pathways at the electrode interfaces, circumventing local film imperfections. This structural compensation is critical for fully exploiting the intrinsic properties of monolayer TMDs in macro-scale devices. This inference can be further supported by our previously published studies on similar WS_2_-based sensor devices [9]. However, for optoelectronic applications requiring high spatial resolution and fast transient responses, IDE architectures are preferred.

#### 3.2.1. Photoconductive

**Figure 7:**
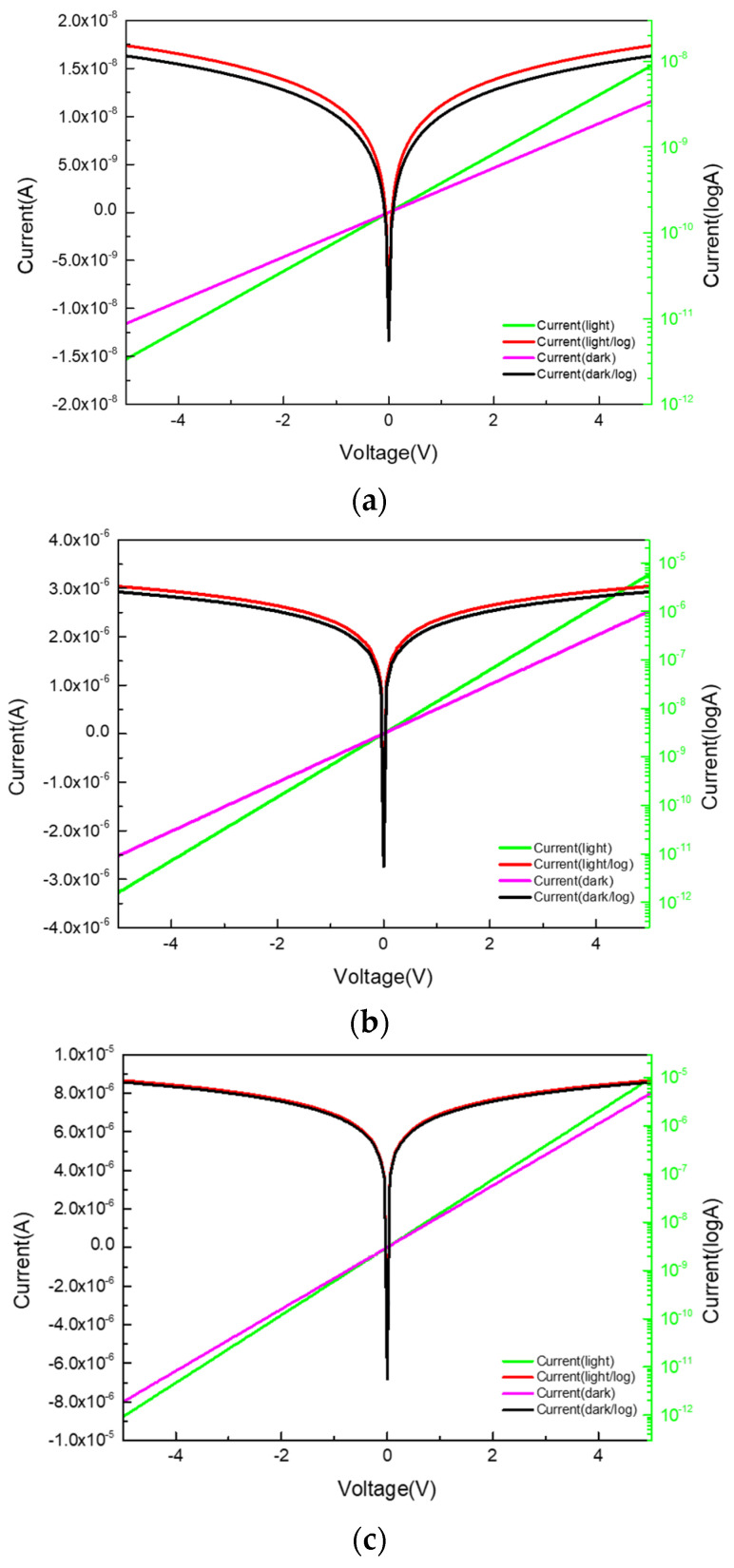
Photocurrent response of (**a**) monolayer, (**b**) bilayer, and (**c**) trilayer MoS_2_ photodetectors under 370 nm illumination.

**Figure 8:**
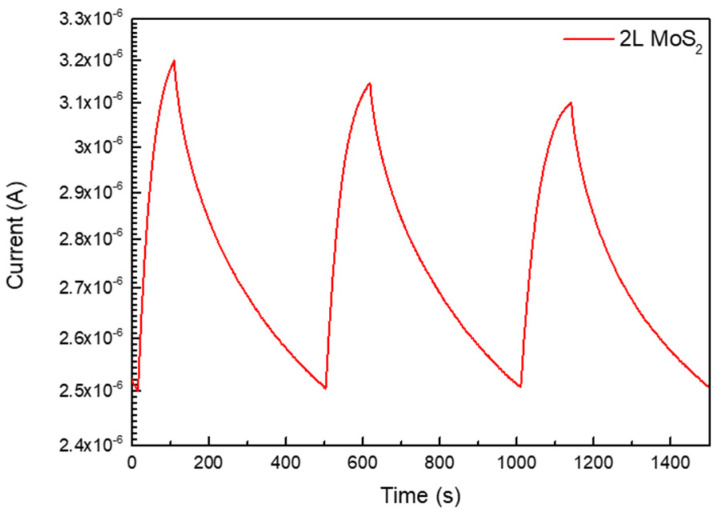
Current-time switching characteristics of the bilayer MoS_2_ photodetector under 370 nm illumination, showing reproducible light-dark switching cycles.

The photoconductive properties of the MoS_2_ photodetectors utilizing IDE architectures were evaluated (Figure 7). The photocurrent of monolayer MoS_2_ was only in the nanoampere range, reflecting the impact of film discontinuity. In contrast, devices with bilayer and trilayer MoS_2_ displayed microampere-level photocurrents, with the bilayer configuration achieving the best balance between film continuity and light-induced current modulation. Under 370 nm illumination at 5 V bias, the bilayer current increased significantly, yielding a responsivity of approximately 7.6 × 10^−6^ A/W. Although this responsivity is lower than values reported for optimized gated MoS_2_ phototransistors, it is reasonable for an unpassivated MSM structure, where the net photocurrent is normalized by the total incident optical power. The relatively low responsivity is attributed to limited absorption in the few-layer channel, trap-assisted recombination at grain boundaries/adsorbates, and contact-limited carrier collection in the lateral IDE geometry. Since Figure 7 already identifies the bilayer device as the best-performing layer configuration, the current-time switching measurement in Figure 8 focuses on the bilayer device to evaluate repeated light-dark switching reproducibility without making the presentation unnecessarily redundant. The bilayer transient response showed reproducible cycling over full light-dark periods. The response and recovery times estimated from the 10–90% rise and 90–10% decay criteria were approximately 70 s and 285 s, respectively. The slow recovery is attributed to adsorbate- and trap-mediated photogating effects in the unpassivated MoS_2_ channel, indicating a slow surface-controlled photoconductive process rather than an intrinsic carrier-transit limitation [6,7,8,9].

#### 3.2.2. Negative Photoconductivity

**Figure 9:**
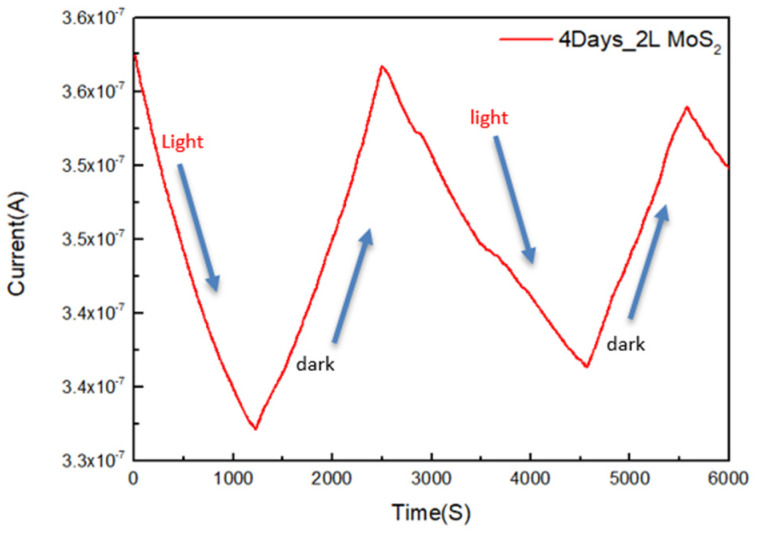
Negative photoconductivity of bilayer MoS_2_ photodetector.

In comparison with our previous results [9] and other reports [7,8,19,20,21], it is evident that while WS_2_ maintained a stable photoresponse, bilayer MoS_2_ demonstrated a high sensitivity to ambient conditions [22,23]. In the present devices, negative photoconductivity (NPC) was most clearly observed in the bilayer MoS_2_ photodetector after days of exposure to ambient conditions, where illumination led to a decrease in current (Figure 9). The bilayer-specific prominence of NPC is attributed to the balance between a continuous conductive channel and strong surface sensitivity to adsorbate-induced trapping. In comparison, the monolayer device is dominated by discontinuous low-current pathways. In contrast, the trilayer device has higher baseline conductance, which may reduce the relative contribution of surface-adsorbate-induced current modulation. When the bilayer device was subsequently measured under vacuum conditions, the NPC was significantly suppressed and gradually diminished, eventually becoming negligible. The observed NPC in bilayer MoS_2_ after ambient exposure is consistent with previous reports [24,25] on environment-sensitive photoresponse in two-dimensional materials. Based on the conclusions reported in these studies on ambient-sensitive photoresponses, a plausible explanation is that surface oxidation and molecular adsorption introduce electron-trapping states [20,21,24,25]. We infer that under illumination, these electron-trapping states capture photogenerated electrons, generating localized charged centers that act as deep-level trap states [20,21]. Consequently, illumination paradoxically decreases the number of free carriers. These oxidized surface groups can further participate in photo-assisted redox reactions with ambient oxygen and moisture. The resulting superoxide and hydroxyl species are adsorbed on the surface of MoS_2_ and continue to capture electrons from the channel, establishing a self-sustaining electron-depletion cycle [24]. The proposed mechanism is supported by the prior literature and is consistent with the observed electrical behavior.

#### 3.2.3. Contact Engineering

**Figure 10:**
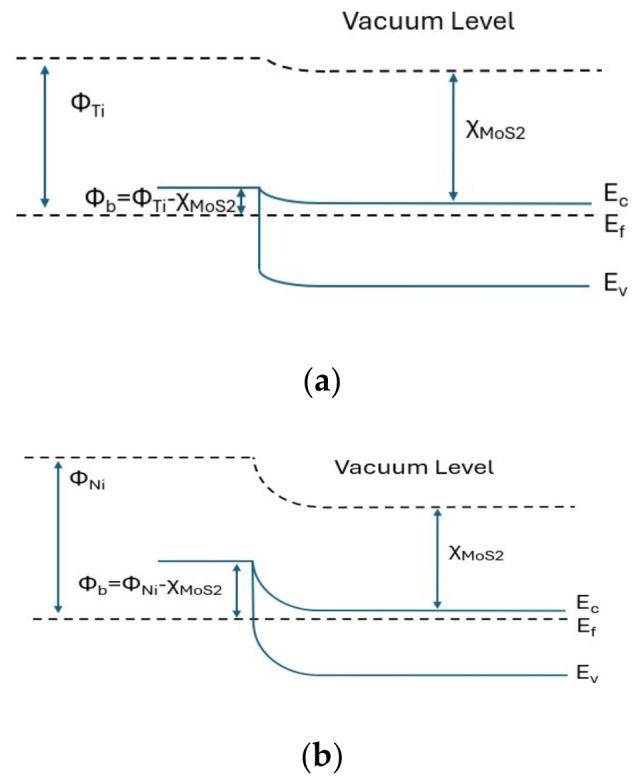
Energy band diagram illustrating ideal alignment between (**a**) Ti and (**b**) Ni contacts and two-dimensional MoS_2_ for forming a near-ohmic contact.

**Figure 11:**
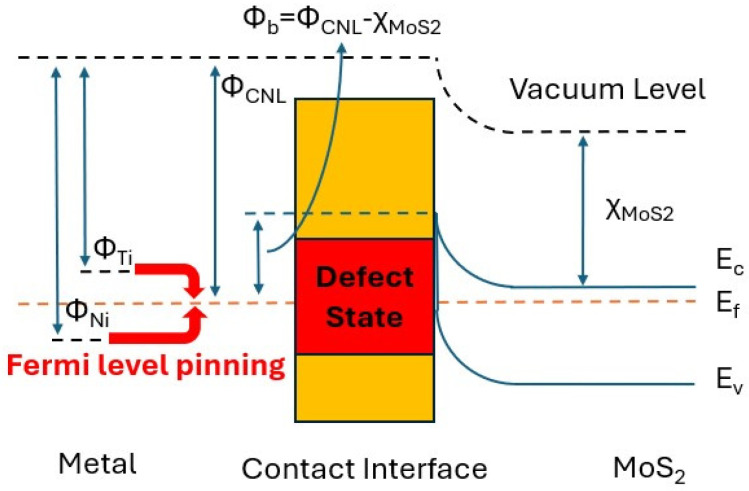
Energy band diagram illustrating Fermi level pinning at the metal/2D MoS_2_ interface.

To optimize device performance, contact engineering was performed through thermal annealing of metal electrodes. Ti and Ni were initially selected based on the ideal band diagram model shown in Figure 10. However, prior to annealing, both contacts exhibited nearly identical electrical characteristics due to the Fermi-level pinning effect at the metal-MoS_2_ interface (Figure 11). In this case, interface states effectively pin the Fermi level, rendering conventional work-function matching ineffective.

**Figure 12:**
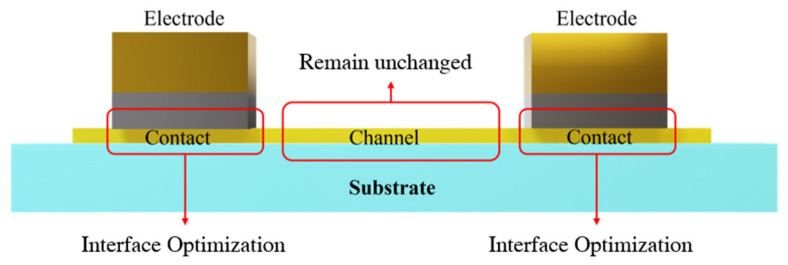
Ni-contact optimization schematic.

**Figure 13:**
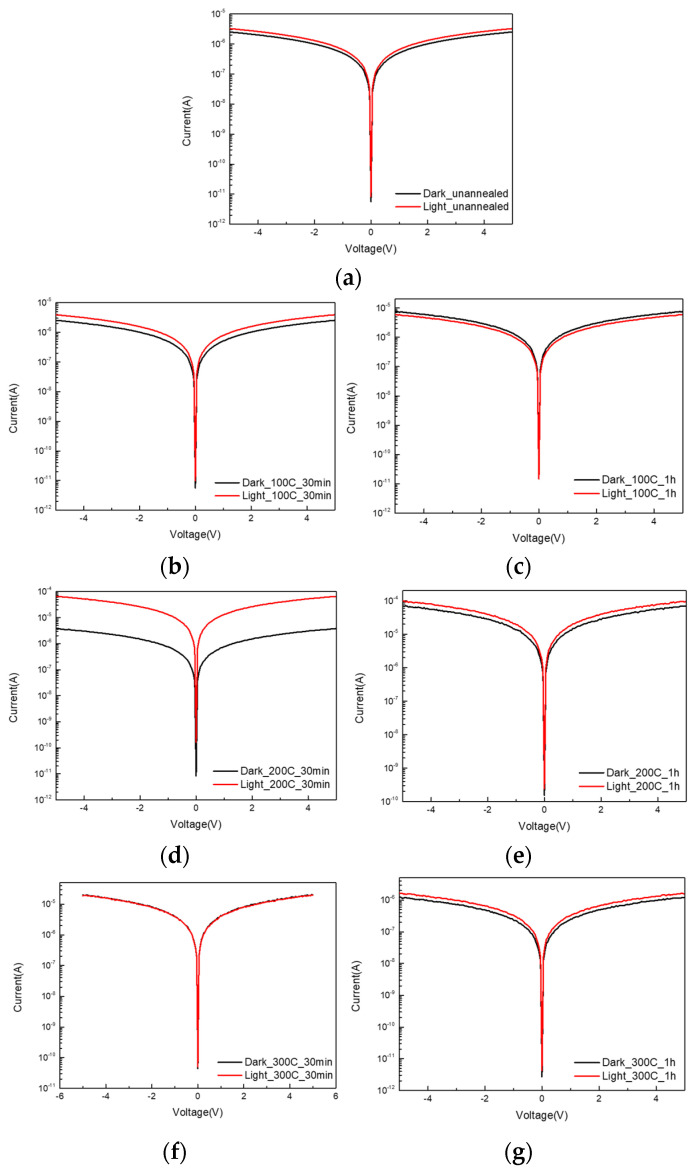
Photocurrent response of bilayer MoS_2_ photodetectors under different annealing conditions: (**a**) as-fabricated, (**b**) 100 °C for 30 min, (**c**) 100 °C for 1 h, (**d**) 200 °C for 30 min, (**e**) 200 °C for 1 h, (**f**) 300 °C for 30 min, and (**g**) 300 °C for 1 h. The improvement is discussed as an effective two-terminal device/contact-related enhancement, as dedicated TLM structures were not fabricated.

To address this limitation, thermal annealing has been widely recognized as an effective strategy to minimize interfacial defects and enhance carrier injection in MoS_2_ devices [21,26]. This process can mitigate contact-related barriers by promoting atomic-scale rearrangement and removing fabrication residues at the metal-semiconductor interface [26]. A localized thermal annealing strategy was adopted (Figure 12). Increasing the annealing temperature up to 200 °C for 30 min led to a pronounced enhancement in photocurrent (Figure 13), indicating improved carrier injection and reduced effective device/contact-related resistance. Because the present photodetectors were fabricated using a fixed IDE geometry rather than dedicated transfer length method (TLM) structures with varied channel spacings, an independent extraction of the contact resistance Rc is not available in this study. Therefore, the annealing effect is discussed in terms of effective two-terminal device improvement rather than a TLM-derived Rc value.

**Figure 14:**
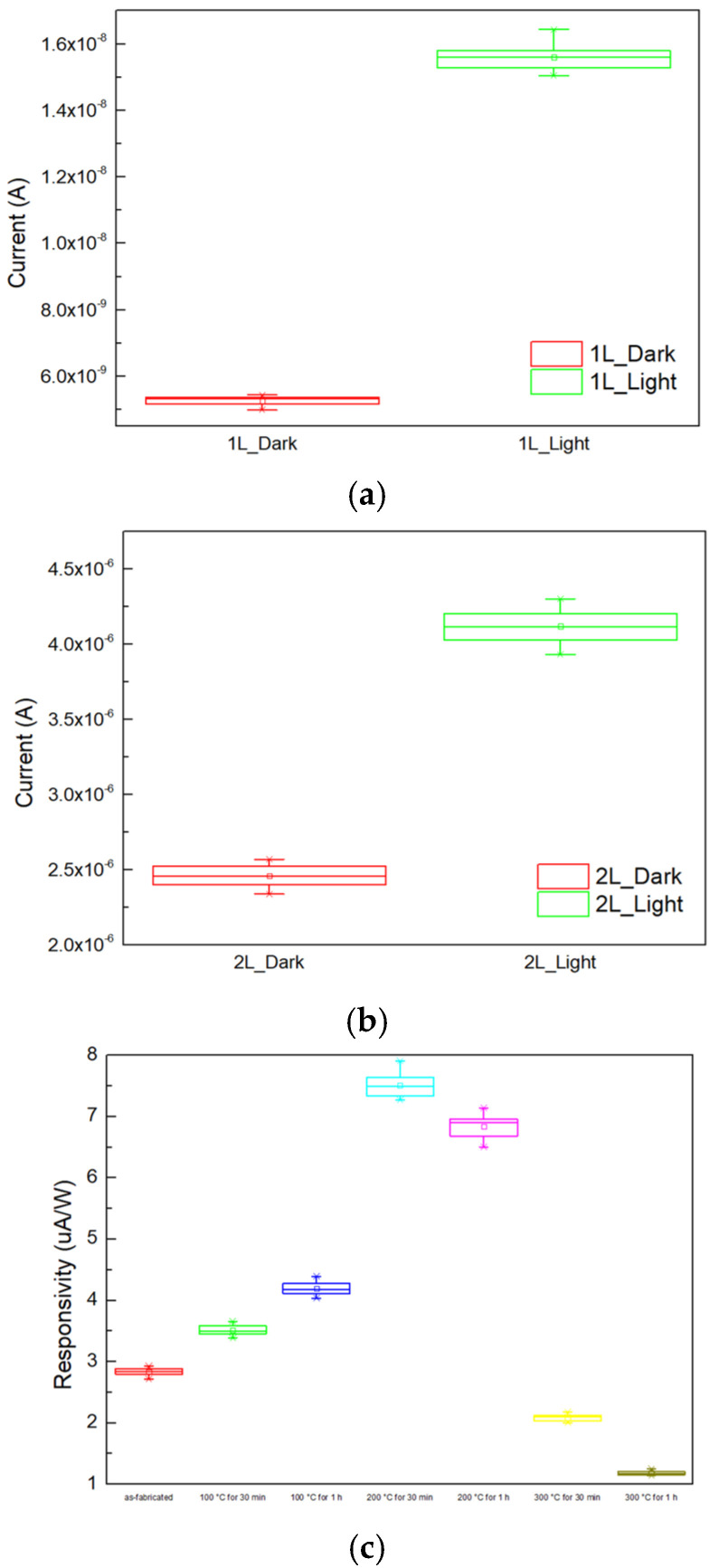
Distribution of dark and light current for (**a**) monolayer and (**b**) bilayer MoS_2_ photodetectors. (**c**) Responsivity distribution of bilayer MoS_2_ photodetectors under various annealing conditions.

However, excessive annealing (e.g., 300 °C) resulted in degradation of the On/Off ratio, likely due to interfacial reactions and structural damage [27], highlighting the importance of optimizing annealing conditions. The presented curves represent typical device behavior, and similar trends were consistently observed across multiple devices, with device-to-device variation within a reasonable range. To evaluate the uniformity and reproducibility of the proposed fabrication process, a total of 49 discrete devices were fabricated and characterized on a single substrate for each sample. After excluding a few devices that were compromised by mechanical damage or manual handling errors during the measurement process, the statistical analysis reveals exceptional consistency across the array. The variations in photoelectric parameters were within a narrow range of ±5% for each sample, as shown in Figure 14. This high degree of uniformity underscores the precision of the oxide-sulfidation technique and its potential for large-scale integration of 2D MoS_2_-based optoelectronics.

## 4. Conclusions

In summary, this study demonstrates a controllable oxide-sulfidation approach for fabricating thickness-tunable few-layer MoS_2_ photodetectors on sapphire substrates. The combined material analyses confirm the successful formation of monolayer, bilayer, and trilayer MoS_2_ films, with few-layer films exhibiting improved continuity and surface quality compared with monolayer MoS_2_. The device results reveal a clear layer-dependent photoresponse, with the bilayer MoS_2_ photodetector showing the best balance between film continuity and light-induced current modulation. In addition, ambient-induced negative photoconductivity was observed in bilayer MoS_2_ and is attributed to surface adsorption and trap-mediated photogating effects. Contact engineering through mild thermal annealing of Ni electrodes further enhanced the photocurrent by improving effective carrier injection at the metal–semiconductor interface. These findings provide a unified understanding of how film thickness, surface adsorption, and interfacial contact properties jointly govern carrier transport and photoresponse in oxide-sulfidized MoS_2_ films, offering practical guidelines for scalable two-dimensional optoelectronic devices.

## Figures and Tables

**Table 1:** Characteristic Raman phonon-mode peaks and peak separations for different layers of MoS_2_.

Layer Number	E_2_g^1^ (cm^−1^)	A_1_g (cm^−1^)	Δ(A_1_g − E_2_g^1^) (cm^−1^)
1	386.36	405.55	19.19
2	385.35	406.06	20.71
3	384.84	406.56	21.72

## Data Availability

The original contributions presented in this study are included in the article. Further inquiries can be directed to the corresponding authors.

## Abbreviations

The following abbreviations are used in this manuscript:

2D	Two-dimensional
TMDs	Transition metal dichalcogenides
MoS_2_	Molybdenum disulfide
WS_2_	Tungsten disulfide
CVD	Chemical vapor deposition
RF	Radio frequency
TMO	Transition metal oxide
MSM	Metal–Semiconductor–Metal
IDEs	Interdigitated electrodes
TEM	Transmission electron microscopy
OM	Optical microscopy
SHG	Second-harmonic generation
EDS	Energy dispersive spectroscopy
MoO_3_	Molybdenum trioxide
NPC	negative photoconductivity
